# Supplementary material for: A Meta-Analysis on the Effect of Dexamethasone on the Sugammadex Reversal of Rocuronium-Induced Neuromuscular Block
Source: J Clin Med. 2020 Apr 24;9(4):1240. doi: 10.3390/jcm9041240 (PMC7230323; doi:10.3390/jcm9041240)
Supplement: Supplementary file 1 [file jcm-09-01240-s001.pdf]

Journal of
Clinical Medicine

MDPI

Article

A Meta-Analysis on the Effect of Dexamethasone on the Sugammadex Reversal of Rocuronium-Induced Neuromuscular Block

Chang-Hoon Koo[1,†], Jin-Young Hwang[2,3,†], Seong-Won Min[2,3,‡,*] and Jung-Hee Ryu[1,3,‡,*]

1 Department of Anesthesiology & Pain medicine, Seoul National University Bundang Hospital, Seongnam 13620, Korea; vollock9@snubh.org
2 Department of Anesthesiology & Pain medicine, SMG-SNU Boramae Medical Center, Seoul 07061, Korea; mistyblue15@naver.com (J.-Y.H.); swmin@snu.ac.kr (S.-W.M.)
3 Department of Anesthesiology & Pain medicine, Seoul National University College of Medicine, Seoul 03080, Korea
† These two authors equally contributed to this work as co-first authors.
‡ These two authors equally contributed to this work as co-corresponding authors.
* Correspondence: swmin@snu.ac.kr (S.-W.M.); rjh74@snu.ac.kr (J.-H.R.); Tel.: +82-2-870-2518 (S.-W.M.); +82-31-787-7497 (J.-H.R.); Fax: +82-2-870-3863 (S.-W.M.); +82-31-787-4063 (J.-H.R.)

Received: 1 April 2020; Accepted: 21 April 2020; Published: 24 April 2020

Abstract: Sugammadex reverses the rocuronium-induced neuromuscular block by trapping the cyclopentanoperhydrophenanthrene ring of rocuronium. Dexamethasone shares the same steroidal structure with rocuronium. The purpose of this study was to evaluate the influence of dexamethasone on neuromuscular reversal of sugammadex after general anesthesia. Electronic databases were searched to identify all trials investigating the effect of dexamethasone on neuromuscular reversal of sugammadex after general anesthesia. The primary outcome was time for neuromuscular reversal, defined as the time to reach a Train-of-Four (TOF) ratio of 0.9 after sugammadex administration. The secondary outcome was the time to extubation after sugammadex administration. The mean difference (MD) and 95% CI were used for these continuous variables. Six trials were identified; a total of 329 patients were included. The analyses indicated that dexamethasone did not influence the time for neuromuscular reversal of sugammadex (MD –3.28, 95% CI –36.56 to 29.99, $p = 0.847$) and time to extubation (MD 25.99, 95% CI –4.32 to 56.31, $p = 0.093$) after general anesthesia. The results indicate that dexamethasone did not influence the neuromuscular reversal of sugammadex in patients after general anesthesia. Therefore, the dexamethasone does not appear to interfere with reversal of neuromuscular blockade with sugammadex in patients undergoing general anesthesia for elective surgery.

Keywords: dexamethasone; neuromuscular blockade; sugammadex

1. Introduction

The prevention of residual neuromuscular blockade (NMB) after general anesthesia is important in decreasing the risk of critical respiratory events [1]. Sugammadex, a selective neuromuscular blocking agent (NMBA)-binding drug, specifically reverses NMB induced by an aminosteroid NMBA, such as rocuronium and vecuronium [2]. Sugammadex is a modified γ-cyclodextrin specifically designed to encapsulate the aminosteroid NMBA, forming a complex with aminosteroid NMBA [2]. This ultimately reduces the concentration of NMBA in the neuromuscular junction and results in a rapid and effective reversal of NMB of any level.

Perioperative dexamethasone has wide applications in treating many clinical conditions with inflammatory response during surgery, such as postoperative hyper-reactive airway [3], anaphylaxis [4], pain [5], and postoperative nausea and vomiting (PONV) [6]. Dexamethasone, as a corticosteroid, shares the same cyclopentanoperhydrophenanthrene structure with aminosteroid NMBA; these structural characteristics of corticosteroid may antagonize the binding of sugammadex with aminosteroid NMBA [7]. Therefore, the possible influence of dexamethasone on the NMB reversal action of sugammadex has been attributed to the structural similarity between aminosteroid muscle relaxants and corticosteroid [7]. An in vitro study reported that dexamethasone inhibited sugammadex NMB reversal activity in a dose-dependent manner in innervated primary human muscle cells [8]. However, the clinical impact of dexamethasone on the effect of sugammadex is still controversial in many clinical studies [9–14]. Therefore, the aim of this meta-analysis was to explore whether the interaction of dexamethasone with sugammadex would lead to a clinical impact on the reversal of NMB. The primary outcome was the difference in the time to recovery of Train-of-Four (TOF) ratio > 0.9 and the secondary outcome was the difference in the time to extubation in patients undergoing general anesthesia where sugammadex was used to reverse rocuronium-induced NMB.

2. Methods

We reviewed the articles according to Preferred Reporting Items for Systematic Reviews and Meta-Analyses (PRISMA) statement guidelines [15] and registered the protocol at the International Prospective Register of Systematic Reviews (registration number: CRD 42018115748).

2.1. Data Sources and Search Strategy (Literature Search)

We searched literature investigating the effect of dexamethasone on the reversal of neuromuscular blockade by sugammadex from the following electronic database: MEDLINE, EMBASE, the Cochrane Central Register of Controlled trials (CENTRAL), Cumulative Index of Nursing and Allied Health Literature (CINAHL), Scopus, Web of Science and KoreaMed. The last search was performed on January 2020. Relevant search terms relating to sugammadex were composed of the medical subject headings (MeSH), text words and controlled vocabulary terms. Results were combined by the Boolean operator "AND" or "OR" with search terms. We applied a specific search strategy for each database (Table S1). There were no restrictions related to language and publication year.

2.2. Study Selection

Initially, two authors (C.-H.K., J.-Y.H.) independently screened the titles and abstracts to include relevant reports and exclude irrelevant reports. Subsequently, full texts of relevant reports were investigated to determine whether they were appropriate for this study. If the two authors' selections did not coincide, the disagreement was arbitrated by consensus with another author (S.-W.M.). If no agreement could be reached despite of the discussion, the final decision was made by J.-H.R.

2.3. Data Extraction and Collection

Two authors (C.-H.K., J.-Y.H.) independently extracted and collected data including study related data (first author, publication year, study design, group, sample size), baseline patient characteristics (age) and variables of interest (dexamethasone doses, type and level of neuromuscular blockade, sugammadex doses) and outcomes (time to recovery, time to extubation). Any discrepancies reached an agreement by consensus with another author (S.-W.M.). If no agreement could be reached despite the discussion, the final decision was made by J.-H.R.

2.4. Methodological Quality and Risk of Bias Assessment

Two authors (C.-H.K., J.-Y.H.) independently assessed the methodological quality and risk of bias of the selected studies. We used Cochrane Collaboration's tool for assessing risk of bias for randomized controlled trials (RCT) [16], and the Newcastle-Ottawa scale for non-randomized

controlled studies of intervention (NRSI) [17]. The Cochrane tool consists of selection bias, performance and detection bias, attrition bias, reporting bias and other forms of bias. Each domain could be categorized as low, unclear, and high. The Newcastle-Ottawa scale is composed of 8 questions, and subdivided into 3 categorizes; selection, comparability and outcome or exposure. Disagreements were resolved by consensus with another author (S.-W.M.). If no agreement was reached despite of the discussion, the final decision was made by J.H.R.

2.5. Outcomes Assessed

The primary outcome was defined as the difference between groups in time to recovery of TOF ratio > 0.9 after sugammadex administration. The secondary outcome was the difference between two groups in time to extubation after sugammadex administration.

2.6. Data Synthesis and Statistical Analyses (Meta-Analysis)

Since the outcomes in this analysis were continuous variables, we (C.-H.K., J.-Y.H.) calculated mean differences (MD) and 95% confidence intervals (CI). Data synthesis and analysis was conducted using R version 3.6.1 (R Foundation for Statistical Computing, Vienna, Austria) [18] with 'meta' package [19]. The findings were presented by forest plot with 95% CIs. I^2 statistic estimated the degree of heterogeneity among the studies. It was interpreted as low ($0 < I^2 < 50\%$), moderate ($50\% \leq I^2 < 75\%$) or high ($I^2 \geq 75\%$). A fixed-effect model was used in case of low level of heterogeneity ($I^2 < 50\%$), otherwise a random-effect model was used. Subgroup analysis was performed based on the study design (RCT vs. NRSI). Sensitivity analysis was conducted to evaluate the influence of a single trial on the pooled effect size by excluding one study. Meta-regression analysis was used to investigate the potential effect modifiers such as study design, mean ages, dexamethasone doses, sugammadex doses, and TOF count before sugammadex administration. Funnel plot was constructed to evaluate symmetrical shape and access publication bias.

3. Results

3.1. Characteristic of Trials and Patients

We retrieved 2457 potentially eligible reports published up to January 2020 by searching electronic databases. Among them, 1056 reports were excluded due to duplicated searches. Subsequently, 1384 and another nine reports were regarded as absolute irrelevant studies by examining titles and abstracts, respectively. A full text of eight studies was reviewed and two records were excluded because one of them was a review article and the other was an in vitro study. Therefore, a total of six full-text studies with 329 patients were included in the final analysis (Figure 1) [9–14]. Characteristics and details of trials are shown in Table 1.

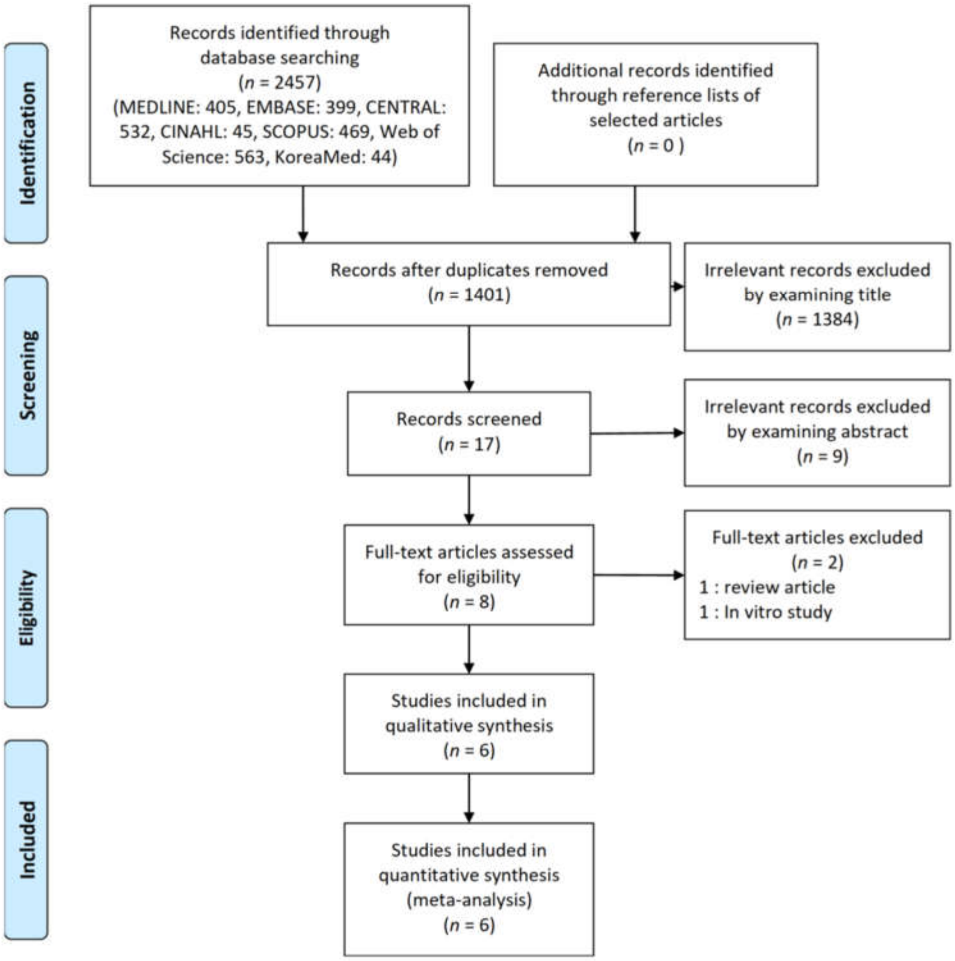

Figure 1. Flow diagram of included and excluded studies.

Table 1. Baseline characteristics and population of the included trials (n = 6).

Study	Design	Size DEX/Control	Age (year) DEX/Control	DEX dose	NMBA		Sugammadex dose
					Type	Level	
Batistaki 2019[9]	RCT	22/22	53.45/52.18	5 mg	ROC	PTC 1-2	4 mg/kg
Buonanno 2016[10]	NRSI	30/15	47/48	8 mg	ROC	TOF 2	2 mg/kg
Gulec 2016[11]	RCT	30/30	5.6/5.2	0.5 mg/kg	ROC	TOF 2	2 mg/kg
Ozer 2018[12]	NRSI	10/30	47/55.62	8 mg	ROC	TOF 1-2	2 mg/kg
Rezonja 2016[13]	RCT	30/30	63/62	0.15 mg/kg	ROC	Desired	200 mg
Saleh 2017[14]	RCT	40/40	2.85/3.40	0.5 mg/kg	ROC	TOF 1	2 mg/kg

Age is expressed as the mean or median values. RCT = randomized controlled trial, NRSI = Non-randomized controlled studies of intervention, DEX = dexamethaxone, NMBA = Neuromuscular blocking agents, ROC = rocuronium, PTC = post-tetanic count, TOF = Train-of-Four.

3.2. Methodological Quality and Risk of Bias

According to the protocol of the previous investigation [20], a different tool was applied for assessing methodological quality and risk of bias based on the study design. We found four RCTs and two NRSIs. The results of the four RCTs are shown in Figure S1. Details of judgement for each trial are represented in Table S2. Most of the risk of bias was assigned to low grade in all studies. However, one study scored "high" in terms of the risk of performance and detection bias because of the single-blinded design [13]. The results of the two NRSIs are summarized in Table S3 with details of judgement. All studies scored 7–8 of 9 points, indicating good quality. We determined not to use a funnel plot and not to evaluate publication bias because of the small number of studies [21].

3.3. Outcome Synthesis

3.3.1. Time to Recovery of TOF Ratio > 0.9

Time to recovery of TOF ratio > 0.9 was reported in six studies, including 329 patients (Figure 2) [9–14]. Patients in the intervention group were administered dexamethasone (*n* = 10) or prednisolone (*n* = 20) in one study [12], and we only extracted data from patients receiving dexamethasone. There was no difference in time to recovery of TOF ratio > 0.9 between the two groups (MD –3.28, 95% CI -36.66 to 29.99, *p* = 0.847). A high level of heterogeneity among the studies was found (I^2 = 94%, *p* < 0.01). Subgroup analysis also showed that there was no difference in both RCT (MD –19.86, 95% CI –2.29 to 42.01, I^2 = 83%, *p* = 0.079) and NRSI subgroups (MD –50.22, 95% CI –104.55 to 4.12, I^2 = 83%, *p* = 0.61).

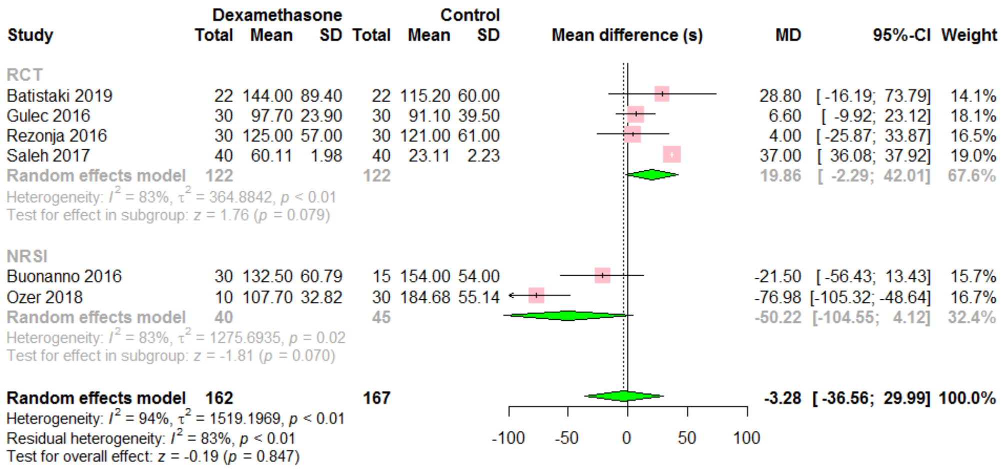

Figure 2. Time(s) to recovery of TOF ratio > 0.9. Dexamethasone vs. control

In the sensitivity analyses, exclusion of any single trial did not change the significance of the result, and the results remained consistent across the different analysis (Figure 3). The covariates included in the meta-analysis were study design, mean ages, dexamethasone dose (mg/kg), sugammadex doses (mg/kg) and TOF count before sugammadex administration. One study missed reporting the mean weight of patients [12] and the missing value was imputed by average weight from other trials with adults [9,10,13]. Finally, meta-regression showed that study design had a significant influence on the pooled effect size (*p* = 0.015), accounting for the high level of heterogeneity (70.83%). According to the result of the meta-regression, dexamethasone reduced the time to recovery in NRSI but increased it in RCTs. Table 2 summarizes the results of the meta-regression analysis.

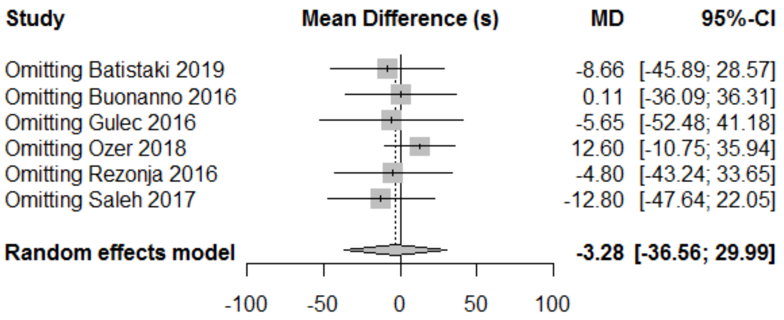

Figure 3. Sensitivity analysis by excluding one trial at time(s) to recovery of TOF ratio > 0.9

Table 2. Meta-regression for the potential sources of heterogeneity.

Variances	Coefficient	Standard error	95% CI	*p* value
Study design	71.0	22.4	27.2 to 114.9	0.001
Mean ages	-0.7	0.6	-1.8 to 0.5	0.250
Dexamethasone dose	102.7	67.5	-29.5 to 234.9	0.128
Sugammadex dose	20.4	25.6	-29.7 to 70.5	0.425
TOF count	-12.5	26.6	-64.7 to 39.7	0.639

3.3.2. Time to Extubation

Time to extubation was reported in three studies including 184 patients (Figure 4) [9,11,14]. There was no difference in time to extubation between groups (MD 25.99, 95% CI –4.32 to 56.31, p = 0.093), with high level of heterogeneity among the studies (I^2 = 90%, p < 0.01).

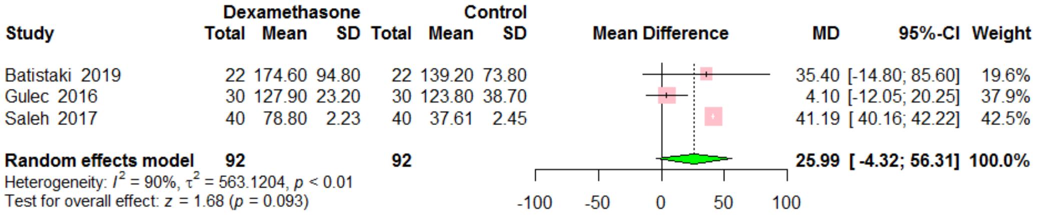

Figure 4. Time to extubation. Dexamethasone administration vs. control.

4. Discussion

The result of this analysis suggests that the use of dexamethasone may not interfere with the reversal of rocuronium-induced NMB with sugammadex; time to recovery of TOF ratio > 0.9 and time to extubation were not delayed by dexamethasone administration before sugammadex reversal of rocuronium-induced NMB.

Sugammadex has a ring-shaped, lipophilic central cavity, encapsulating the cyclopentanoperhydrophenanthrene ring of rocuronium within its cavity [22]. Dexamethasone shares the same steroidal structure with rocuronium [7]. Thus, concerns regarding the possible interaction between sugammadex and dexamethasone have been raised. Theoretically, two types of drug interaction—displacement and capturing—may occur with sugammadex.

Another drug may bind to sugammadex by displacing rocuronium from sugammadex, resulting in a recurrence of NMB. Sugammadex may also bind to a third drug instead of rocuronium as a capturing interaction, decreasing the efficacy of sugammadex towards rocuronium [23]. However, our review and meta-analysis revealed that the reversal of rocuronium-induced NMB with

sugammadex was not affected by dexamethasone. Possible explanations are based on the selective high affinity of rocuronium for sugammadex and the stability of the rocuronium-sugammadex complex. Sugammadex, a modified γ-cyclodextrin by adding eight-sided chains at the typical chemical structure of cyclodextrin, enlarges the central cavity to be the most appropriate cavity size for rocuronium, allowing greater and selective encapsulation of rocuronium [22]. Rocuronium has been reported to have a greater affinity for sugammadex binding than corticosteroids [24,25]. According to in vitro studies using isothermal titration calorimetry by Zhang et al. [24], over 40 lipophilic steroid and non-steroid drugs are able to undergo displacement interactions with sugammadex; however, the affinities for these are 120- to 700-fold lower than that for rocuronium. Zwiers et al. [25] used pharmacokinetic–pharmacodynamic modelling to evaluate the possible interactions between sugammadex and 300 commonly prescribed drugs, including corticosteroids. The result showed that only three drugs (toremifene, fusidic acid, and flucloxacilin) have the potential to displace rocuronium from sugammadex among all of the tested drugs [25]. Rocuronium had a 10,000-fold greater affinity than dexamethasone, and about 100- to 500-fold greater affinity than other corticosteroids for sugammadex binding. Rocuronium enters the central cavity of sugammadex, forming a stable and tight complex via strong intermolecular forces, hydrogen bonds, and electrostatic interaction [26], and, therefore, rocuronium is rarely detached with sugammadex. Sugammadex may bind to hormonal contraceptives [25,27], but the interactions of sugammadex with other drugs have not been reported to date.

Meta-regression analysis showed that the study design significantly influenced the mean differences of the time to recovery of TOF ratio > 0.9. Subgroup analysis also indicated that the study design influenced the result of this study. Dexamethasone administration tended to shorten time to recovery of TOF ratio > 0.9 in the NSRI subgroup whereas dexamethasone administration tended to prolong time to recovery of TOF ratio > 0.9 in the RCT subgroup, although statistical significance was not reached in the subgroup analysis. However, these findings need to be interpreted with caution due to a small number of included studies. Further studies may be needed.

It should be noted that the published studies included in this review and meta-analysis were performed with a single dose of dexamethasone and mostly under moderate NMB. Thus, the effects of chronic steroid therapy on sugammadex reversal of rocuronium-induced NMB and the drug interaction between sugammadex and corticosteroid under deep NMB still remain unclear.

5. Conclusions

In conclusion, the use of dexamethasone does not interfere with the reversal of rocuronium-induced NMB with sugammadex. Considering the limitations of this study, further studies are necessary.

Supplementary Materials: The following are available online at www.mdpi.com/2077-0383/9/4/1240/s1, Table S1: Search strategy for each database, Table S2: Details for judgement for each risk of bias for randomized controlled studies, Table S3: Details for judgement of each quality assessment for non-randomized controlled studies. Figure S1: Risk of bias summary and graph.

Author Contributions: Conceptualization, C.-H.K., and J.-H.R.; methodology, C.-H.K., J.-Y.H., S.-W.M. and J.-H.R.; validation, J.-H.R.; formal analysis, C.-H.K. and J.-Y.H.; investigation, C.-H.K. and J.-Y.H. ; data curation, C.-H.K., J.-Y.H., S.-W.M. and J.-H.R.; writing - original draft preparation, C.-H.K. and J.-Y.H.; writing - review & editing, S.-W.M. and J.-H.R. visualization, C.-H.K.; supervision, J.-H.R. All authors have read and agreed to the published version of the manuscript.

Funding: This research received no external funding.

Conflicts of Interest: The authors declare no conflict of interest.

References

1. Sauer, M.; Stahn, A.; Soltesz, S.; Noeldge-Schomburg, G.; Mencke, T. The influence of residual neuromuscular block on the incidence of critical respiratory events. A randomised, prospective, placebo-controlled trial. *Eur. J. Anaesthesiol.* **2011**, *28*, 842–848.
2. Suy, K.; Morias, K.; Cammu, G.; Hans, P.; van Duijnhoven, W.G.; Heeringa, M.; Demeyer, I. Effective reversal of moderate rocuronium- or vecuronium-induced neuromuscular block with sugammadex, a selective relaxant binding agent. *Anesthesiology* **2007**, *106*, 283–288.
3. Cross, K.P.; Paul, R.I.; Goldman, R.D. Single-dose dexamethasone for mild-to-moderate asthma exacerbations: Efficacy, easy, and accepatable. *Can. Fam. Physician* **2011**, *57*, 1134–1136.
4. Liyanage, C.K.; Galappatthy, P.; Seneviratne, S.L. Corticosteroids in management of anaphylaxis; a systemic review of evidence. *Eur. Ann. Allergy Clin. Immunol.* **2017**, *49*, 196–207.
5. De Oliveira, G.S., Jr.; Almeida, M.D.; Benzon, H.T.; McCarthy, R.J. Perioperative single dose systemic dexamethasone for postoperative pain: A meta-analysis of randomized controlled trials. *Anesthesiology* **2011**, *115*, 575–588.
6. De Oliveira, G.S. Jr.; Castro-Alves, L.J.; Ahmad, S.; Kendall, M.C.; McCarthy, R.J. Dexamethasone to prevent postoperative nausea and vomiting: An updated meta-analysis of randomized controlled trials. *Anesth. Analg.* **2013**, *116*, 58–74.
7. Mirakhur, R.K. Sugammadex in clinical practice. *Anaesthesia* **2009**, *64* (Suppl. 1), 45–54.
8. Rezonja, K.; Sostaric, M.; Vidmar, G.; Mars, T. Dexamethasone produces dose-dependent inhibition of sugammadex reversal in in vitro innervated primary human muscle cells. *Anesth. Analg.* **2014**, *118*, 755–763.
9. Batistaki, C.; Pandazi, A.; Kyttari, A.; Kaminiotis, E.; Kostopanagiotou, G. Is there an interaction between dexamethasone and sugammadex in real clinical conditions? A randomized controlled trial in patients undergoing laparoscopic cholecystectomy. *J. Anaesthesiol. Clin. Pharmacol.* **2019**, *35*, 215–219.
10. Buonanno, P.; Laiola, A.; Palumbo, C.; Spinelli, G.; Servillo, G.; Di Minno, R.M.; Cafiero, T.; Di lorio, C. Dexamethasone Does Not Inhibit Sugammadex Reversal After Rocuronium-Induced Neuromuscular Block. *Anesth. Analg.* **2016**, *122*, 1826–1830.
11. Gulec, E.; Biricik, E.; Turktan, M.; Hatipoglu, Z.; Unlugenc, H. The Effect of Intravenous Dexamethasone on Sugammadex Reversal Time in Children Undergoing Adenotonsillectomy. *Anesth. Analg.* **2016**, *122*, 1147–1152.
12. Ozer, A.B.; Bolat, E.; Erhan, O.L.; Kilinc, M.; Demirel, I.; Toprak, G.C. Sugammadex Improves Neuromuscular Function in Patients Receiving Perioperative Steroids. *Niger. J. Clin. Pract.* **2018**, *21*, 139–142.
13. Rezonja, K.; Mars, T.; Jerin, A.; Kozelj, G.; Pozar-Lukanovic, N.; Sostaric, M. Dexamethasone does not diminish sugammadex reversal of neuromuscular block—Clinical study in surgical patients undergoing general anesthesia. *BMC Anesthesiol.* **2016**, *16*, 101.
14. Saleh, R.S.; Moustafa, M.A. Recovery from rocuronium with sugammadex in children premedicated with dexamethasone for prevention of postoperative nausea and vomiting. *Egypt. J. Anaesth.* **2017**, *33*, 1–4.
15. Moher, D.; Liberati, A.; Tetzlaff, J.; Altman, D.G. Preferred reporting items for systematic reviews and meta-analyses: The PRISMA statement. *J. Clin. Epidemiol.* **2009**, *62*, 1006–1012.
16. Higgins, J.P.; Altman, D.G.; Gotzsche, P.C.; Juni, P.; Moher, D.; Oxman, A.D.; Savovic, J.; Schulz, K.F.; Weeks, L.; Sterne, J.A. The Cochrane Collaboration's tool for assessing risk of bias in randomised trials. *BMJ* **2011**, *343*, d5928.
17. Wells, G.A.; Shea, B.; O'Connell, D.; Peterson, J.; Welch, V.; Losos, M.; Tugwell, P. The Newcastle-Ottawa Scale (NOS) for Assessing the Quality of Nonrandomised Studies in Meta-Analyses. Available online: www.ohri.ca/programs/clinical_epidemiology/oxford.asp (accessed on 10 February, 2020).
18. R Core Team. A language and environment for statistical computing. R Foundation for Statistical Computing, Vienna, Austria. Available online: https://www.R-project.org (accessed on 11 March 2020)
19. Schwarzer, G. Meta: An R package for meta-analysis. *R News*. **2007**, 7, 40-45.
20. Faber, T.; Ravaud, P.; Riveros, C.; Perrodeau, E.; Dechartres, A. Meta-analyses including non-randomized studies of therapeutic interventions: A methodological review. *BMC Med. Res. Methodol.* **2016**, *16*, 35.
21. Egger, M.; Davey, S.G.; Schneider, M.; Minder, C. Bias in meta-analysis detected by a simple, graphical test. *BMJ* **1997**, *315*, 629–634.

22. Akha, A.S.; Rosa, J., 3rd; Jahr, J.S.; Li, A.; Kiai, K. Sugammadex: Cyclodextrins, development of selective binding agents, pharmacology, clinical development, and future directions. *Anesthesiol. Clin.* **2010**, *28*, 691–708.
23. Rex, C.; Bergner, U.A.; Puhringer, F.K. Sugammadex: A selective relaxant-binding agent providing rapid reversal. *Curr. Opin. Anaesthesiol.* **2010**, *23*, 461–465.
24. Zhang, M.Q. Drug-specific cyclodextrins: The future of rapdid neuromuscular block reversal. *Drugs Future* **2003**, *28*, 347.
25. Zwiers, A.; van den Heuvel, M.; Smeets, J.; Rutherford, S. Assessment of the potential for displacement interactions with sugammadex: A pharmacokinetic-pharmacodynamic modelling approach. *Clin. Drug Investig.* **2011**, *31*, 101–111.
26. Adam, J.M.; Bennett, D.J.; Bom, A.; Clark, J.K.; Feilden, H.; Hutchinson, E.J.; Palin, R.; Prosser, A.; Rees, D.C.; Rosair, G.M.; et al. Cyclodextrin-derived host molecules as reversal agents for the neuromuscular blocker rocuronium bromide: Synthesis and structure-activity relationships. *J. Med. Chem.* **2002**, *45*, 1806–1816.
27. Cada, D.J.; Levien, T.L.; Baker, D.E. Sugammadex. *Hosp. Pharm.* **2016**, *51*, 585–596.

© 2020 by the authors. Licensee MDPI, Basel, Switzerland. This article is an open access article distributed under the terms and conditions of the Creative Commons Attribution (CC BY) license (http://creativecommons.org/licenses/by/4.0/).
